# Electromagnetic metamaterials to approach superconductive-like electrical conductivity

**DOI:** 10.1038/s41598-023-29966-2

**Published:** 2023-02-24

**Authors:** A. Danisi, C. Zannini

**Affiliations:** 1 CERN, Geneva, Switzerland; 2 Present Address: ITER, Saint-Paul-Lez-Durance, France

**Keywords:** Physics, Metamaterials, Electrical and electronic engineering

## Abstract

This paper explores the possibility of using electromagnetic metamaterials to synthesize an equivalent structure that approaches superconductive-like properties, i.e. extremely high electrical conductivity. The underlying electromagnetic model is formalized analytically using transmission line theory and supported by experimental measurements. This particular use of metamaterials could bring to the ground-breaking scenario of developing superconductive-like cavities and lossless guiding structures at ambient temperature.

## Introduction

Because of their interesting properties, the proposal of using metamaterials^1–3^ for electromagnetic applications such as antennas^4^, optical and quasi-optical transformations^5,6^, and even particle accelerators^7,8^ is found in literature.

Metamaterials are interesting because they exhibit, under certain circumstances, negative electrical permittivity and/or negative magnetic permeability. This property enables the proposal of a variety of electromagnetic designs which are particularly intriguing, e.g. propagation of waves below cut-off in a waveguide, above-cut-off stop-band in a waveguide, backward-wave propagation, optical and quasi-optical cloaking^1^, antenna performance optimization^4^, impedance mitigation in particle accelerators^7,8^.

In this paper, the possibility to adopt a properly engineered metamaterial to equivalently synthesize a perfect electrical conductor, i.e. replicating superconductive-like conditions, is investigated. In more detail, the possibility to cancel the surface impedance of a resistive wall by interposing a layer of metamaterial is hereafter addressed.

In transmission lines and waveguides, the possibility to decrease the Radio-Frequency (RF) resistive losses below the theoretical limit of usual copper losses, without making use of superconductors, is explored in the concept of corrugated waveguides^9–11^. In these waveguides, the resistive walls are modulated with periodic narrow-band structures (i.e. lambda-quarter lines) simulating open-circuit conditions, which allows an improved loss performance^12^. However, this solution reduces the losses at the expenses of the propagating mode, which is not anymore either a TE or a TM mode, but rather a hybrid mode. In addition, the corrugation is intrinsically narrow-band and poses limitations on the operational frequency range^12^. Finally, the waveguide geometry is certainly more complex, and requires tight manufacturing tolerances for the fabrication at higher frequencies^9–12^.

Strictly speaking, to make its impedance vanish, the resistive wall has to behave like a perfect electric conductive material (PEC). This implies infinite equivalent conductivity and, therefore, zero equivalent surface impedance. A pipe with this special wall would behave like an ideal guiding structure, allowing wave propagation without losses. Likewise, a confined volume with the same special wall would behave as an ideal RF cavity. This behaviour is currently achieved by means of the use of superconductive materials. It is well known that these materials require cryogenic systems to keep their temperature below the critical limit value^13^.

On this basis, the usefulness of synthesizing a perfect electrical conductor at room temperature and at relatively wide bandwidth, which is the objective of the work presented in this paper, reveals to be scientifically and technologically attractive.

The paper is organised as follows: section named "Theoretical Analysis" describes the analytical treatment of the electromagnetic problem. The underlying equations can be used to properly design an equivalent loss-less structure in a certain frequency range; section named "Experimental Observations" reports the experimental measurements already presented and analysed in^8^ which support the analytical findings; the last section outlines the potentially beneficial impact of this theory on currently relevant technologies where RF losses represent a significant design issue.

## Theoretical analysis

### Transmission-line equivalence

**Figure 1:**
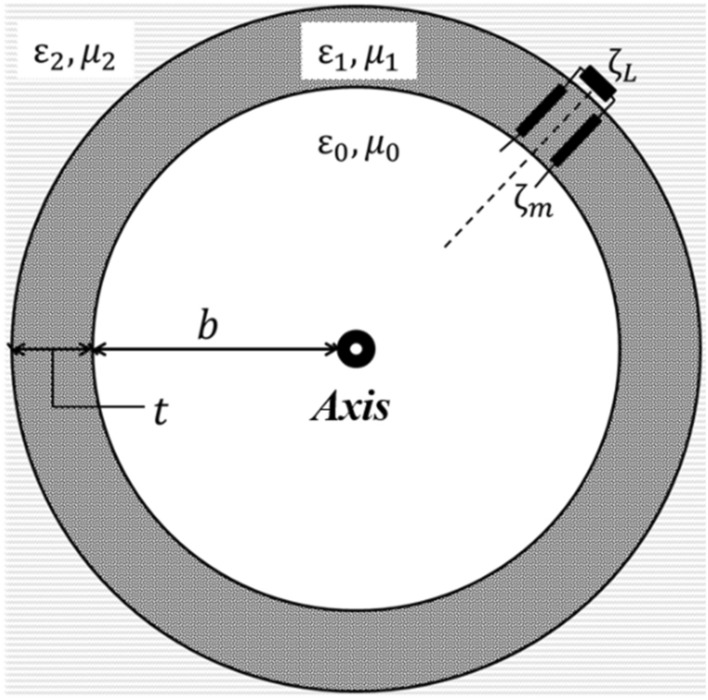
Transverse geometry of the problem. The metamaterial layer is put between the vacuum and the surrounding wall, which extends to infinity.

The theoretical analysis focuses on the case where a layer of metamaterial is applied on the resistive wall of a waveguide structure, as in Fig. 1. In this framework, the calculation of the equivalent surface impedance seen by the internal space, i.e. the surface impedance of the resistive wall “modified” by the interposing layer, can be performed using the transmission line formalism.

More in detail^14^, this equivalent impedance can be calculated, under certain hypotheses, as the surface impedance of the resistive wall transformed along a transmission line of length *t* (with reference to Fig. 1), using the common transmission line impedance formula^15^.

The underlying hypotheses to adopt this approach are the following:

- The geometry must be so that cylindrical wave attenuation can be neglected: with reference to Fig. 1, this translates to the following condition
  1 $$\begin{gathered} \delta \ll b \hfill \\ t \ll b \hfill \\ \end{gathered}$$

where $\delta$ is the penetration depth of the electromagnetic field in the metal wall. Under this hypothesis, the analogy of multi-layer structures with transmission line sections is valid, provided that normal incidence to the layer is satisfied.

- The material properties are such that the permittivity and permeability of the first layer satisfy
  2 $$\left| {\varepsilon_{1} \mu_{1} } \right| \gg \varepsilon_{0} \mu_{0}$$
  where ${\varepsilon }_{0}$ and ${\mu }_{0}$ denote vacuum permittivity and permeability. Similarly, ${\varepsilon }_{1}$ and ${\mu }_{1}$ are the complex permittivity and permeability of the interposed metamaterial layer (Fig. 1). Under this assumption^16^, the equivalent surface impedance calculated transforming the wall impedance across a transmission line, is independent from the incidence angle of the wave.

Therefore, under the above-mentioned assumptions, regardless of the incidence of the electromagnetic wave, it is possible to calculate the equivalent impedance of the resistive wall using the simple transmission line model as depicted in Fig. 1, with normal incidence.

In this equivalence, the transmission line can be considered closed on a surface impedance defined by

3 $$\zeta_{L} = \frac{1 + j}{{\sigma_{2} \delta }}$$

where ${\sigma }_{2}$ is the wall conductivity. In this framework, an appropriate estimation of the penetration depth is obtained adopting the well-known expression valid for good conductors

4 $$\delta = \sqrt {\frac{2}{{\omega \sigma_{2} \mu_{2} }}}$$

where ${\mu }_{2}$ is the wall permeability, as per Fig. 1, and $\omega =2\pi f$ is the angular frequency.

### Generic derivation of the transformed impedance

With reference to Fig. 1 and hypothesis (1), the problem becomes a relatively simple condition of guided-wave propagation. The wave in the vacuum volume will “see” an equivalent impedance at the boundary with the metamaterial layer. This equivalent impedance corresponds to the surface impedance (previously defined), transformed along the equivalent transmission line of the metamaterial layer. In symbols, ${\zeta }_{m}={\text{T}}\left({\zeta }_{L}\right)$, where ${\text{T}}$ denotes the impedance transformation function, operated on the load impedance (in this case, the surface impedance ${\zeta }_{L}$). In a transmission line, the impedance transformation along a certain line of length $t$ can be formalised using the transmission line impedance formula^15^, yielding for this case

5 $${\zeta }_{m}={\zeta }_{1}\cdot \frac{{\zeta }_{L}+j{\zeta }_{1}\mathrm{tan}{k}_{1}t}{{\zeta }_{1}+j{\zeta }_{L}\mathrm{tan}{k}_{1}t}$$

where ${\zeta }_{1}=\sqrt{{\mu }_{1}/{\varepsilon }_{1}}$ is the characteristic impedance of the metamaterial insertion, ${k}_{1}=\omega \sqrt{{\mu }_{1}{\varepsilon }_{1}}$ is the related propagation factor, $t$ is the line length (layer thickness, as in Fig. 1).

At this point, some observations need to be pointed out, for the treatment not to lose generality and, at the same time, to allow adequate simplification:

- Given that the wall surface impedance is of the form given by Eq. (3), the following notation can be adopted: ${\zeta }_{L}=a+ja$, with $a$ being a real and positive quantity; it is worth mentioning that this hypothesis is valid, for good conductors, until optical frequencies or above;
- The characteristic impedance of the metamaterial insertion is in principle complex, i.e. $\zeta_{1} = b^{\prime} + jb^{\prime\prime}$; note that no assumption is made on the sign or magnitude of either real or imaginary part of this impedance;
- As in any generic treatment of transmission line theory, the propagation factor is also considered complex; in particular, being always multiplied by the thickness in Eq. (5), the following notation can be adopted: ${k}_{1}t=\beta -j\alpha$, with $t$ being real and positive.

As a direct consequence of observation c), the tangent in Eq. (5) can be developed further, taking into account the following equivalence with hyperbolic functions^17^

6 $$\mathrm{tan}\left(\beta -j\alpha \right)=\frac{\mathrm{tan}\,\beta -j\,\mathrm{tan\,h\,}\alpha }{1+j\,\mathrm{tan}\,\beta\, \mathrm{tan\,h}\,\alpha }$$

Adopting the notations described above and the equivalence in (6), Eq. (5) can be written in expanded form and subsequently rationalised, to extract real and imaginary parts.

For the sake of this paper, only the real part is the objective of the analytical treatment, since it corresponds to the resistive part of the transformed impedance, i.e. the impedance “seen” by the wave in the vacuum volume.

Before proceeding further, a brief justification is needed to why the imaginary part is hereby not interesting. Contrary to what happens to a purely real load, an imaginary load at the end of a transmission line can always be compensated with an element having equal and opposite reactance (e.g. a stub closed on a short circuit, a lumped-element reactance). The interest of the present paper lies in investigating the possibility to cancel the real part, without really posing any condition on the imaginary part. In fact, considering a null real part, the load would have a purely imaginary impedance. As such, a purely imaginary load will always be characterized by a unitary reflection coefficient (in absolute value) with respect to a lossless line. It would exhibit a phase shift, which is however unimportant.

By virtue of the aforementioned observations, the real part of the transformed impedance is therefore analytically calculated as

7 $$Re\left\{ {\zeta_{m} } \right\} = \frac{{\left( {b^{{\prime}{2}} + b^{{\prime\prime}{2}} } \right) \cdot N_{1} }}{D} + \frac{{\left( {b^{{\prime}{2}} - b^{{\prime\prime}{2}} + 2b^{\prime}b^{\prime\prime}} \right) \cdot N_{2} }}{D} + \frac{{\left( {b^{{\prime}{3}} + b^{\prime}b^{{\prime\prime}{2}} } \right) \cdot N_{3} }}{D} + \frac{{\left( {b^{{\prime\prime}{3}} + b^{{\prime}{2}} b^{\prime\prime}} \right) \cdot N_{4} }}{D} + \frac{{N_{5} }}{D}$$

where

8 $$\begin{gathered} D = \left| {b^{\prime} - b^{\prime\prime}\tan \beta \tanh \alpha - a\left( {\tan \beta - \tanh \alpha } \right) + j\left[ {a\left( {\tan \beta + \tanh \alpha } \right) + b^{\prime}\tan \beta \tanh \alpha + b^{\prime\prime}} \right]} \right|^{2} \hfill \\ N_{1} = a\left[ {1 + \left( {\tan \beta } \right)^{2} \left( {\tanh \alpha } \right)^{2} } \right] + b^{\prime}\tanh \alpha - b^{\prime\prime}\tan \beta \hfill \\ N_{2} = a\left[ {\left( {\tan \beta } \right)^{2} + \left( {\tanh \alpha } \right)^{2} } \right] \hfill \\ N_{3} = \tanh \alpha \left( {\tan \beta } \right)^{2} \hfill \\ N_{4} = \tan \beta \left( {\tanh \alpha } \right)^{2} \hfill \\ N_{5} = 2a^{2} b^{\prime}\tanh \alpha \left[ {1 + \left( {\tan \beta } \right)^{2} } \right] + 2a^{2} b^{\prime\prime}\tan \beta \left[ {1 - \left( {\tanh \alpha } \right)^{2} } \right] \hfill \\ \end{gathered}$$

Equation (7) is quite general: it can be used for the extraction of the real part of a transformed impedance along any line length $t$, for any frequency $f$, any interposing material properties (captured by ${\mu }_{1}$ and ${\varepsilon }_{1}$, which can be complex and/or negative), as long as observation a) is valid. This means that (7) can be used whenever it is needed to calculate the equivalent impedance of a metal when a coating layer of thickness $t$ is applied on top of it.

Before proceeding with the application of (7) to a metamaterial interposing layer, a thorough benchmarking of the analytical formula has to be performed, in order to assess the consistency and coherence of (7) with respect to known scenarios. The known scenarios that shall be used are those which give rise to known results, i.e. the case of open and short loads, as well as the matched load case, all of them considered in a loss-less line.

For a short-circuit load in a loss-less line, $\alpha = 0$, $a = 0$, $b^{\prime\prime} = 0$, and therefore the coefficients given in (8) reduce to the following list

9 $$\begin{gathered} D = \left| {b^{\prime}} \right|^{2} \hfill \\ N_{1} = 0 \hfill \\ N_{2} = 0 \hfill \\ N_{3} = 0 \hfill \\ N_{4} = 0 \hfill \\ N_{5} = 0 \hfill \\ \end{gathered}$$

and (7) becomes

10 $$Re\left\{ {\zeta_{m} } \right\} = 0$$

which is in full agreement with transmission line theory: the transport of a short-circuit load along a line always gives rise to a purely imaginary impedance.

Similar is the case of an open-circuit load in a loss-less line, whose analysis is skipped for the sake of conciseness ($\alpha = 0$, $a \to \infty$, $b^{\prime\prime} = 0$).

The case of a matched load yields to $b^{\prime\prime} = b^{\prime} = a$. This means that the line has to be necessarily considered as a lossy line. In this case, analytical manipulations of (7) and (8), with due consideration of hypotheses a)-c), lead to

11 $$Re\left\{ {\zeta_{m} } \right\} = a$$

which is consistent with transmission line theory for a matched line.

The consistency of the found Eqs. (7) and (8) has been therefore successfully benchmarked against the open, short and matched scenarios. For completeness, the derivation of Eq. (7), as well as the benchmarking against the short, open and matched conditions have been successfully verified with the symbolic calculator Mathematica®.

### Calculation of the transformed impedance for metamaterial layer

Equation (7) gives now the analytical basis on which the calculation of the transformation of a metal wall impedance along a metamaterial layer can be performed. A practical example (illustrated in the values of Table 1) can be considered as a case study, yet fully synthesizable, e.g. see^18^.

**Table 1:** Parameters for Case study.

Parameter	Value
Metal conductivity	${2\cdot 10}^{7}$ S/m
Metamaterial type	ENG (Negative electrical permittivity)
${\varepsilon }_{r1}$	$-10$ (lossless case); $-10+j{10}^{-4}$ (lossy case) $-10+j{10}^{-5}$ (lossy case)
${\mu }_{r1}$	1
Metamaterial layer thickness	10 mm

**Figure 2:**
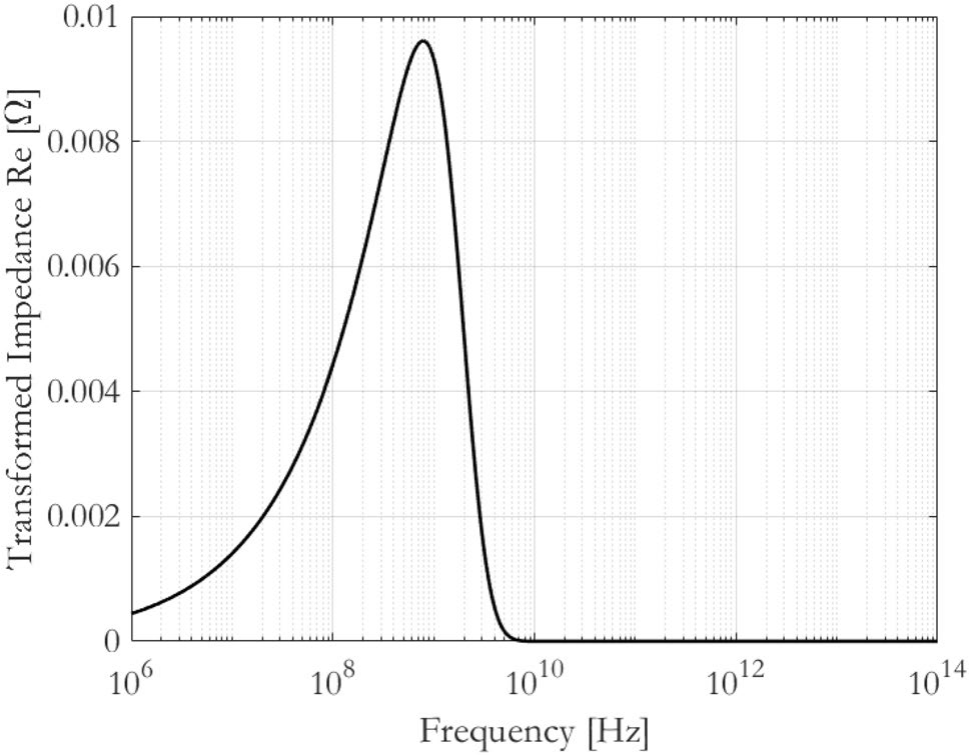
Real part of transformed impedance according to values in Table 1. Lossless case considered (i.e. real and negative value of electrical permittivity).

The values listed in Table 1 represent a rather generic example, where a good conductor (such as Molybdenum, Tungsten or Brass) is “masked” with a 1-cm-thick layer of negative-permittivity (ENG) metamaterial. With these illustrative values, Eq. (7) can be used to predict and plot the real part of the transformed impedance. The results are depicted in Fig. 2.

From these results, it is evident that above a certain frequency, the material starts to exhibit an equivalent behaviour of a Perfect Electrical Conductor (PEC), i.e. equivalent resistance approaching zero.

In fact, the usual dependence of the surface impedance on frequency is following the definition (3), which imposes an increasing trend. Instead, Fig. 2 shows that, when approaching a certain frequency, this trend changes: the dependence defined in (3) is reversed by the metamaterial insertion, giving rise to a sort of transition. The frequency value around which this “metaconductive” transition happens, depends on material parameters, as well as engineering factors such as thickness and material manufacturing dimensions. However, it is clear from Fig. 2 that the use of such metamaterials beyond the metaconductive transition would be beneficial, whenever the needs to have extremely low resistive losses are important.

It is useful to notice that for frequencies reasonably beyond the metaconductive transition, Fig. 2 shows a perfect cancellation of the transformed resistance. This behaviour is maintained by virtue of the “lossless” hypothesis of the metamaterial insertion, i.e. the relative permittivity, even if negative, is purely real.

A more realistic model of an equivalent metamaterial would actually require to also take into account the imaginary part of the relative permittivity.

As to the value of such imaginary part, it is meaningful to consider reasonably small values, since this part is normally associated to loss phenomena. The latter are commonly designed to be minimized, and therefore the imaginary part of the relative permittivity is hereby chosen to assume the values in Table 1, for comparison with the lossless case. These values correspond to very low loss metamaterials, yet considered to be fully synthesizable, e.g.^18^.

**Figure 3:**
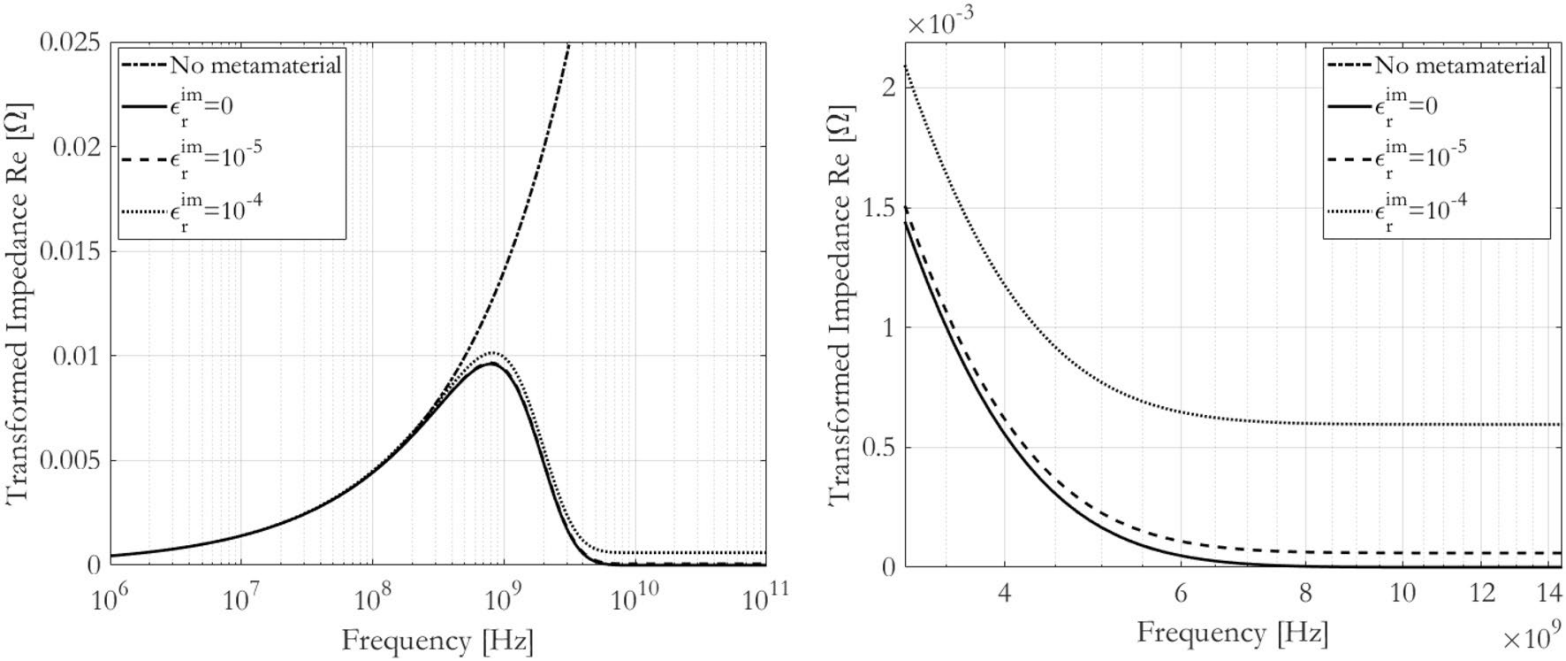
(Left) Real part of transformed impedance according to values in Table 1 for lossy cases. (Right) Zoom on a reduced frequency range, beyond the metaconductive transition.

Figure 3 shows the effect of finite imaginary relative permittivity on the metaconductive transition of the transformed real part of the impedance. It is useful to notice that, for this particular case, values of ${10}^{-4}$ are already giving rise to significant decrease of the equivalent resistance, above the metaconductive transition happening at about 2 GHz. For example, for a frequency of 10 GHz the equivalent resistance is reduced by about two orders of magnitude. With even lower losses, e.g. values of ${10}^{-5}$, the behaviour is found to even closely approach the lossless curve. It is likewise interesting to notice that, above the metaconductive transition, the equivalent resistance appears to be frequency-independent and directly related to the metamaterial losses. However, its nature of being frequency-independent is due to the fact that in Table 1 the imaginary part of the electrical permittivity is specified as frequency-independent.

Therefore, Fig. 3 shows that although it is clear that to perfectly nullify the transformed real part of the impedance it is necessary to have a lossless metamaterial, it is also clear that approaching this nullification can be envisaged minimizing the metamaterial losses.

### Simplified design equation for metaconductive surfaces

Given the complete analytical treatment so far presented, it may be useful to provide a first-term design equation set to identify possible candidate materials for a certain application, or fix some engineering factors (e.g. thickness). Such design equation can help a potential electromagnetic designer to start conceiving the correct metamaterial structure to achieve metaconductive behaviour.

To this aim, it is convenient to write Eq. (7) in a simplified way, e.g. assuming no dielectric and magnetic losses. In these hypotheses, one can find that the transformed real part of the impedance is given by

12 $$Re\left\{ {\zeta_{m} } \right\} = \zeta_{1} \cdot \frac{{A\left[ {1 + \left( {\tan k_{1} t} \right)^{2} } \right]}}{{\left( {A - \tan k_{1} t} \right)^{2} + \left( {\tan k_{1} t} \right)^{2} }}$$

where

13 $$A = \sigma_{2} \delta \zeta_{1}$$

From the latter equations it can be seen that the equivalent behaviour of a PEC structure can be synthesized by simply imposing the following condition

14 $$\tan \left( {k_{1} t} \right) = j$$

This condition is fulfilled only if ${k}_{1}t$ is purely imaginary, which translates into the condition

15 $$\varepsilon_{1} \cdot \mu_{1} < 0$$

This last condition can be satisfied exclusively with the use of a metamaterial, either of ENG (Negative electrical permittivity—${\varepsilon }_{1}<0$ and ${\mu }_{1}>0$) or MNG (Negative magnetic permeability—${\varepsilon }_{1}>0$ and ${\mu }_{1}<0$) type. In this case, Eq. (14) can be rewritten as

16 $$\tanh \left( {\left| {k_{1} t} \right|} \right) = 1$$

This condition is mathematically satisfied when $\left|{k}_{1}t\right|\to \infty$. However, the condition is practically reached (with an absolute error of less than ${10}^{-8}$) for

17 $$\left| {k_{1} t} \right| \ge 10$$

It is worth reminding that, according to what has been found so far, ${k}_{1}$ is purely imaginary, and ${\varepsilon }_{1}$, ${\mu }_{1}$ are purely real, satisfying condition (15).

Equation (17) is the main design rule for metaconductive surfaces. Taking into account the definition of ${k}_{1}$ for a metamaterial satisfying (15), Eq. (17) can be rewritten as

18 $$t \sqrt {\left| {\varepsilon_{r1} } \right|} \ge \frac{10 c}{{\omega_{0} \sqrt {\left| {\mu_{r1} } \right|} }}$$

where $c=1/\sqrt{{\mu }_{0}{\varepsilon }_{0}}$ is the speed of light in vacuum.

**Figure 4:**
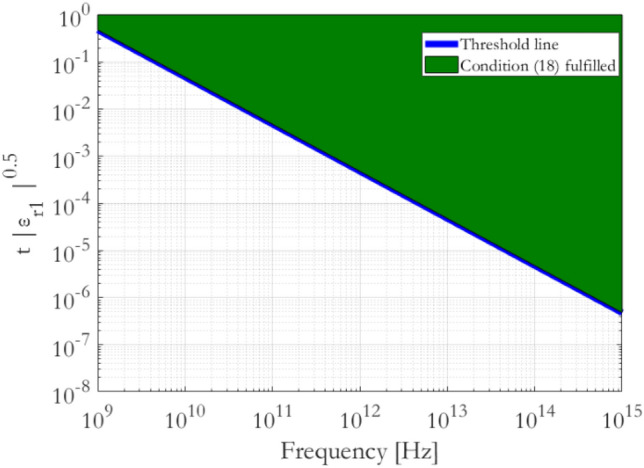
Design space for ENG metaconductive surfaces (${\varepsilon }_{r1}<0$ and ${\mu }_{r1}=1$) according to Eq. (18). Analogous design space can be plotted for MNG metamaterials (${\mu }_{r1}<0$ and ${\varepsilon }_{r1}=1$).

In Eq. (18), $t$ is the needed thickness of the metamaterial layer with relative permittivity ${\varepsilon }_{r1}$ and relative permeability ${\mu }_{r1}$ to design a metaconductive surface for angular frequencies $\omega >{\omega }_{0}$. Therefore, Eq. (18) identifies a design space for the metaconductive surfaces. For example, for ENG metamaterials (${\varepsilon }_{r1}<0$ and ${\mu }_{r1}=1$) this equation is corresponding to Fig. 4. The green zone represents the design space where the pairs $[t, {\varepsilon }_{r1}]$ satisfy (18) and, therefore, give rise to a metaconductive surface.

In reality, synthesized metamaterials exhibit negative-permittivity (ENG) or negative-permeability (MNG) behaviour in narrow frequency bandwidths^1^. Therefore, the metaconductive behaviour will be observed exclusively within these frequency bandwidths, provided that Eq. (18) is thereby satisfied.

To give a practical example of application of the design Eq. (18), a potential metaconductive surface at a frequency of ${\omega }_{0}=2\pi \cdot 14.4$ GHz would require a thickness $t=10$ mm of an ENG metamaterial layer with ${\varepsilon }_{r1}=-10$ and ${\mu }_{r1}=1$ at the frequency ${\omega }_{0}$.

Therefore, for those applications where the equivalent behaviour of a PEC (i.e. superconductive-like performance) is needed in a certain frequency range, Eq. (18) can be used for a preliminary selection and specification of the metaconductive surface. The complete exploration of the physical dependencies is given by Eq. (7) instead.

## Experimental observations

The “metaconductive” behaviour, previously discussed analytically, could be experimentally verified measuring the surface impedance of a structure as that of Fig. 1.

A possible way to perform such a measurement with good resolution is to create a resonant empty cavity in the frequency range of interest, and subsequently measure the unloaded quality factor of the cavity modes. The unloaded quality factor of a resonance associated to an empty cavity is related solely to the losses on the conductive walls. Such losses are due to the surface impedance of the walls, which depends on the material conductivity and frequency, as previously analysed. The quality factor is inversely proportional to the surface resistance^19^. Therefore, for a metal cavity without any metamaterial insertion, the unloaded quality factor is directly related to the electrical conductivity of the metal.

Conversely, for a metal cavity with metamaterial insertion, the above-mentioned analytical treatment predicts that a significant decrease of the surface impedance should be observed in the frequency range where the metamaterial satisfies Eq. (18). This should result in a corresponding increase of the measured unloaded quality factor.

The measurement results and discussions detailed in the next sub-sections confirm the analytical findings.

### Measurements results

In the framework of investigating the use of metamaterial insertions for beam-coupling impedance mitigation, an experimental set-up with a waveguide has been built and presented in^8^, together with the related measurements results.

The cavity has been synthesized using a straight section of a rectangular copper waveguide WR284. The waveguide section has been terminated with a metallic plate on both sides, leaving a small opening for a tiny antenna on one side only. The latter antenna is connected to a Vector Network Analyzer (VNA) through a coaxial cable, and used to excite the cavity modes, measure the scattering parameter and subsequently enable the derivation of the unloaded quality factors for each mode.

The derivation of the unloaded quality factor $Q$ is done considering its definition, as follows

19 $$Q = Q_{L} \left( {1 + 2g} \right)$$

where ${Q}_{L}$ is the loaded quality factor, i.e. the quality factor when the cavity is unavoidably coupled with the measurement setup, and $g$ is the line-cavity coupling factor^15^. The latter is obtained by measuring the Voltage Standing Wave Ratio (VSWR)^15^.

Measurements have been performed with and without metamaterial insertions. The insertions consist of two split-ring resonators (SRRs) stripes on the cavity walls^8^. SRRs show negative relative permeability in a narrow bandwidth, around a well-defined resonance frequency^1^. Therefore, an effect is expected to be observed in this particular frequency bandwidth.

**Figure 5:**
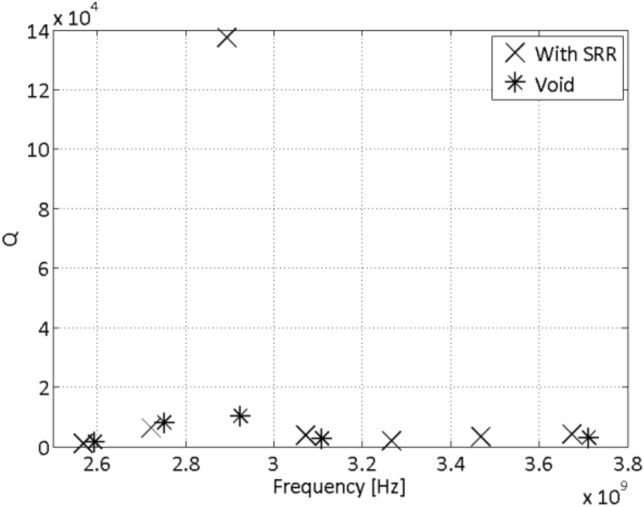
Measured unloaded quality factors (Q) with and without SRR insertions.

The results depicted in Fig. 5 show that at about 2.9 GHz the measured unloaded quality factor is significantly higher in the case of metamaterial presence, meaning a decrease by an order of magnitude in the surface impedance. This important decrease of the surface impedance testifies the metaconductive behaviour of the measured cavity, in accordance with analytical findings.

It is interesting to mention that, since the unloaded quality factor is proportional to the square root of the wall material electrical conductivity ^15^, the increase of a factor 10 on the quality factor observed in Fig. 3 (compared to the bare copper case) corresponds to an equivalent transformed electrical conductivity which is two orders of magnitude higher than that of any normal-conductive material at room temperature.

The other measurement points in Fig. 5 show instead little or no variation when the SRRs are inserted in the waveguide. This does not come as a surprise since both material properties and the condition to get the equivalent behaviour of a perfect electrical conductor are frequency dependent (see Eqs. (7) and (18)).

## Conclusions and outlook

The possibility of obtaining the equivalent behaviour of a perfect electrical conductive wall for an electromagnetic wave by means of metamaterial insertions has been analytically addressed. The required conditions to be satisfied in order to design the appropriate metamaterial insertions (e.g. thickness and material properties) are found analytically by means of a transmission-line model.

Existing experimental measurements conducted on a copper-wall Radio-Frequency cavity with metamaterial insertions have actually shown significant reduction of the resistive losses. This represents an experimental evidence of the analytical predictions presented in this paper, i.e. that a properly-engineered metamaterial insertion could significantly diminish the resistive losses.

The authors consider that the idea proposed in this paper could be a first step towards the fascinating scenario of conceiving equivalent “superconductive-like” structures at room temperature, i.e. without the necessity to employ costly, complex and energy-consuming cryogenic systems to approach the perfect-conductor behaviour. A potential and significant example of such a scenario is the realization of resonant cavities for quantum computing^20,21^.

As an outlook on potential future investigations and researches, topics and areas which will definitely require further analysis are related to the role, limits and minimization of dielectric and magnetic losses in candidate metamaterials^22^. The imaginary part of the transported impedance for practical systems will be explored in future works. In fact, it might be relevant for applications of unidirectional electromagnetic wave control^23,24^.

Further research can be also directed towards experimental characterization, as well as design optimization of metamaterial properties to maximize the metaconductive effect. The views and opinions expressed herein do not necessarily reflect those of the ITER Organization.

## Data Availability

The datasets used and/or analysed during the current study available from the corresponding authors on reasonable request.
